# Head impulse testing in bilateral vestibulopathy in patients with genetically defined CANVAS

**DOI:** 10.1002/brb3.2546

**Published:** 2022-05-02

**Authors:** Max Borsche, Vera Tadic, Inke R. König, Katja Lohmann, Christoph Helmchen, Norbert Brüggemann

**Affiliations:** ^1^ Institute of Neurogenetics University of Lübeck Lübeck Germany; ^2^ Department of Neurology University Medical Center Schleswig‐Holstein Campus Lübeck Lübeck Germany; ^3^ Institute of Medical Biometry and Statistics University of Lübeck Lübeck Germany

**Keywords:** ataxia, bilateral vestibulopathy, CANVAS, RFC1, vestibulo‐ocular reflex, video head impulse test

## Abstract

**Background:**

To investigate the association between disease duration and the severity of bilateral vestibulopathy in individuals with complete or incomplete CANVAS (Cerebellar Ataxia with Neuropathy and Vestibular Areflexia Syndrome) and biallelic *RFC1* repeat expansions.

**Methods:**

Retrospective analysis of clinical data and the vestibulo‐ocular reflex quantified by the video head impulse test in 20 patients with confirmed biallelic *RFC1* repeat expansions.

**Results:**

Vestibulo‐ocular reflex gain at first admittance 6.9 ± 5.0 years after disease onset was 0.16 [0.15–0.31] (median [interquartile range]). Cross‐sectional analysis revealed that gain reduction was associated with disease duration. Follow‐up measurements were available for ten individuals: eight of them exhibited a progressive decrease of the vestibulo‐ocular reflex gain over time. At the first visit, six of all patients (30%) did not show clinical signs of cerebellar ataxia.

**Conclusions:**

Our data suggest a pathological horizontal head impulse test, which can easily be obtained in many outpatient clinics, as a sign of bilateral vestibulopathy in genetically confirmed CANVAS that can precede clinically accessible cerebellar ataxia at least in a subset of patients. The presumably continuous decline over time possibly reflects the neurodegenerative character of the disease. Thus, genetic testing for *RFC1* mutations in (isolated) bilateral vestibulopathy might allow disease detection before the onset of cerebellar signs. Further studies including a wider spectrum of vestibular function tests are warranted in a prospective design.

## Graphical abstract

To investigate the association between bilateral vestibulopathy and disease duration in genetically defined CANVAS, the vestibulo‐ocular reflex was quantified by the video head impulse test in 20 RFC1 repeat expansion carriers. We found a decrease in vestibulo‐ocular reflex gain over time, possibly reflecting the neurodegenerative character of the disease. Furthermore, our data suggest that bilateral vestibulopathy might precede cerebellar ataxia in RFC1‐associated disease, at least in a subset of patients.

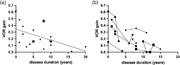

## 1 INTRODUCTION

The genetic cause of CANVAS, a previously clinically defined syndrome of cerebellar ataxia, neuropathy, and bilateral vestibulopathy, was deciphered in 2019 as a biallelic intronic repeat expansion in the *RFC1* gene (Cortese et al., 2019). The mutation is detectable in > 90% of patients with the complete symptom triad (Cortese et al., 2019; Gisatulin et al., 2020). Since then, a few attempts have been performed to describe the natural history of *RFC1*‐positive CANVAS. Their focus was on cerebellar features (Cortese et al., 2020; Traschütz et al., 2021) and neuropathy (Curro et al., 2021), but not on bilateral vestibulopathy. Hypothesizing that the vestibular organ progressively degenerates over the disease course (Ishai et al., 2021), we investigated the association between bilateral vestibulopathy and disease duration in patients with *RFC1*‐positive CANVAS by analyzing the gain reduction of the vestibulo‐ocular reflex quantified by the video head impulse test, a widely accessible method that can easily be obtained in many outpatient clinics.

## 2 METHODS

The group of CANVAS patients comprised 20 carriers of biallelic repeat expansions in the *RFC1* gene recruited at the outpatient clinics for ataxia and vertigo at the Department of Neurology, University Hospital Schleswig‐Holstein, Campus Lübeck, Lübeck, Germany. The retrospective study analyzing anonymized data was approved by the ethics committee of the University of Lübeck. Genetic confirmation of biallelic *RFC1* repeat expansion was performed at the Institute of Neurogenetics, University of Lübeck, Lübeck, Germany, as described (Gisatulin et al., 2020) by a screening step performing duplex PCR analyses followed by fragment length analysis, repeat‐primed PCR, Sanger sequencing, and Southern blotting to determine repeat type and length.

Disease onset was defined by patients reporting to have recognized complaints associated with vestibular or cerebellar dysfunction, or neuropathy such as stand, gait or speech disturbance, limb ataxia, or oculomotor abnormalities for the first time.

Quantitative analysis of the gain of the vestibulo‐ocular reflex by video‐oculography of the horizontal head impulse test was performed as previously described (Helmchen et al., 2017). Only the horizontal vestibulo‐ocular reflex was analyzed. In brief, eye and head movements were recorded by a digital video camera (Eye‐SeeCam HIT System, Autronics, Hamburg, Germany) at a sampling rate of 220 Hz. At least ten passive and rapid (peak velocity 250°/sec) head movements of small amplitude (10–15°) were performed per side. Head impulses were unpredictable for direction and amplitude. The gain of the horizontal vestibulo‐ocular reflex was analyzed at a narrow time interval of 60 ± 10 ms after head movement onset.

A mean gain <0.7 was considered pathologic (Machner et al., 2021; Yip et al., 2016). If more than one video head impulse test result was available, only the first testing was used for a cross‐sectional analysis to focus on the earliest disease stage possible. In seven patients with follow‐up measurements, longitudinal results were analyzed on a descriptive level. The mean vestibulo‐ocular reflex gain used in the present study was assessed by calculating the mean of the gain of the right and the left vestibular organ. Simple linear regression was calculated to assess the association between the mean vestibulo‐ocular reflex gain and disease duration. Analysis were performed and the figure was created with GraphPad Prism 8. Data are available from the corresponding author upon reasonable request.

## 3 RESULTS

We investigated 20 *RFC1*‐positive patients (6 female [30.0 %], age at onset 61.0 ± 7.8 years [mean ± standard deviation], age at examination 67.8 ± 7.6 years, disease duration 6.9 ± 5.0 years), of which 14 had the complete clinical presentation with the triad of bilateral vestibulopathy, cerebellar ataxia, and neuropathy at the time of diagnosis (“Complete CANVAS,” 70.0 %) (Table 1).

**TABLE 1:** Demographics, clinical symptoms, and technical measurements

Number	1	2	3	4	5	6	7	8	9	10	11	12	13	14	15	16	17	18	19	20	
sex	F	F	M	F	M	F	M	M	M	M	M	M	F	M	M	M	F	M	M	M	F:M = 6:14
Age at onset	61	56	59	60	60	69	65	55	59	56	39	62	65	69	68	62	66	66	49	74	61.0 +/‐ 7.8 (mean +/‐ SD)
Age at 1^st^ examination	66	58	69	80	70	73	72	60	60	71	47	72	67	72	69	70	71	74	59	76	67.8 +/‐ 7.6 (mean +/‐ SD)
Disease duration until 1^st^ examination	5	2	10	20	10	4	7	5	1	16	8	10	2	3	1	8	5	8	10	2	6.9 +/‐ 5.0 (mean +/‐ SD)
Stance																					
Unsecure	√	√	x	√	√	√	√	x	x	√	√	√	√	√	√	x	√	√	√	√	16/20 (80 %)
Gait																					
broad‐based/atactic	√	√	x	√	√	√	√	x	x	√	√	√	√	√	√	√	√	√	√	√	17/20 (85 %)
aggravated gait testing impossible	√	√	√	√	√	√	√	x	√	√	√	√	√	√	√	√	√	√	√	√	19/20 (95 %)
Cerebellar signs																					
limb ataxia	√	x	x	x	x	√	√	x	x	√	x	√	√	√	√	√	√	√	x	√	12/20 (60 %)
oculomotor abnormalities																					
saccadic pursuit	√	√	x	√	x	√	√	√	x	√	x	√	x	√	√	√	√	x	√	√	14/20 (70 %)
downbeat nystagmus	√	x	x	√	x	√	√	x	x	√	x	√	x	x	√	√	√	x	√	√	11/20 (55 %)
dysarthria	√	√	x	x	x	√	√	x	x	√	x	√	x	√	√	x	x	√	x	x	9/20 (45 %)
Neuropathy (clinical signs)																					
reduced sense of vibration	√	√	x	√	√	√	√	√	x	√	x	√	√	√	x	√	√	√	√	√	16/20 (80 %)
achilles tendon reflex reduced/absent	√	√	x	√	√	√	√	√	x	√	x	√	√	√	√	√	√	x	√	√	16/20 (80 %)
MRI																					
cerebellar atrophy	NA	x	x	NA	NA	NA	x	NA	x	√	NA	√	NA	√	√	NA	√	x	NA	NA	5/10 (50%)
ENG																					
reduced SNAP upper limbs	NA	NA	NA	NA	√	NA	NA	NA	NA	√	NA	NA	√	√	√	√	√	NA	NA	NA	7/7 (100 %)
Summary																					
complete CANVAS	√	√		√		√	√			√		√		√	√	√	√	√	√	√	14 (60 %)
no cerebellar ataxia					√			√					√								3 (15 %)
vestibulopathy only			√						√		√										3 (15 %)

**Demographics, clinical symptoms and technical measurements**. All participants had an abnormal clinical head impulse test and a mean gain < 0.07 in the video head impulse test; F – female; M – male; Age at onset was defined as first timepoint the patient noticed symptoms of vestibular or cerebellar dysfunction or neuropathy; √ – present; x – absent; limb ataxia was defined by abnormal finger‐nose‐test and/or abnormal heal‐shin‐slide test; MRI ‐ magnetic resonance imaging; ENG – electroneurography; Sensory neuropathy was clinically defined by the presence of reduced sense of vibration and the absence of the achilles deep tendon reflex; ENG was considered pathologic in the case of either a reduction or a loss of the sensory nerve action potential (SNAP) or slowing at the upper limbs using clinically established cut‐off values; diagnosis of cerebellar ataxia was established by clinical examination performed by an experienced neurologist based on cerebellar oculomotor signs, limb ataxia, and/or dysarthria.

The remaining six patients (30 %, one female [16.7 %], age at onset 56.2 ± 9.0 years, age at examination 62.2 ± 8.6 years, disease duration 6.0 ± 4.0 years) did not show cerebellar signs upon clinical investigation and three of them neither cerebellar ataxia nor neuropathy at the first visit. Of those, four of five patients with clinical follow‐up visits (after 3.0 ± 2.0 years) developed cerebellar signs over the disease course.

**FIGURE 1:**
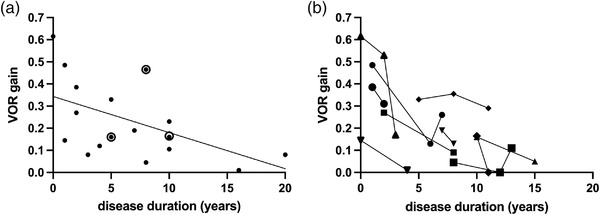
Course of bilateral vestibulopathy in *RFC1* repeat expansion carriers. (a) Relationship between vestibulo‐ocular reflex gain and disease duration cross‐sectionally assessed in 20 biallelic *RFC1* repeat expansion carriers. The line represents linear regression. Circles represent two overlapping data points. (b) Longitudinal measurements of video‐ocular reflex gain and disease duration in ten patients with biallelic repeat expansions in the *RFC1* gene

The mean vestibulo‐ocular reflex gain of both vestibular organs at first presentation was 0.16 [0.15–0.31] (median [interquartile range]). Cross‐sectional investigation of the mean vestibulo‐ocular reflex gain and disease duration suggests an association between both parameters (simple linear regression, *r* ^2^ = .24, *p* = .027, Figure 1A). Repeated video head impulse test measurements, available for 10 subjects, depicted a vestibulo‐ocular reflex gain reduction in 8 of 10 subjects over time (Figure 1B).

## 4 DISCUSSION

IN an attempt to characterize the natural course of bilateral vestibulopathy in CANVAS, we related its most representative and easily accessible sign, that is, the abnormal video head impulse test, of biallelic *RFC1* expansion carriers (all of them with BV) to disease duration and other CANVAS symptoms. In contrast to the recent studies on the clinical phenotype of *RFC1* mutation carriers with various but inconsistent methods of vestibular testing (Cortese et al., 2020; Dominik et al., 2021; Traschütz et al., 2021), we focused on the horizontal high‐frequency vestibulo‐ocular reflex quantified by video head impulse testing as it is applicable in many outpatient clinics.

Three of the 20 patients displayed isolated bilateral vestibulopathy and one‐third of the examined patients did not suffer from cerebellar ataxia at the first visit albeit an average disease duration of 7 years, representing a similar distribution compared to CANVAS cohorts published to date (Cortese et al., 2020; Dominik et al., 2021; Traschütz et al., 2021). Of note, due to the lack of nerve conduction studies at the time point of the first examination, the presence of neuropathy had to be defined in most cases by clinical signs only. However, as most patients without cerebellar signs at first presentation developed cerebellar ataxia over time, our data suggest that bilateral vestibulopathy might precede the other characteristic clinical disease signs of CANVAS, at least in a subset of patients.

We demonstrate a profoundly reduced vestibulo‐ocular reflex gain in *RFC1* expansion carriers already at the first visit, which is in clear contrast to healthy individuals, in whom the vestibulo‐ocular reflex gain remains largely stable even in the very old population (McGarvie et al., 2015). Moreover, our investigations revealed a decline of vestibular function associated with increasing disease duration. These findings were in line with longitudinal data from a subset of patients and together might indicate that progressive neurodegeneration in CANVAS affects not only the cerebellum but also the vestibular organ/nerve. The longitudinal data, albeit only available for a subset of patients, suggest that also the easily accessible horizontal video head impulse can be used to monitor disease progression on an individual level. Moreover, future studies investigating the video head impulse test longitudinally with a larger sample size might contribute to models predicting disease progression. Limitations of our study consist of relatively small sample size, particularly regarding the longitudinal data, the retrospective design, and the analysis of the horizontal vestibulo‐ocular reflex only, which, however, is easily accessible in many outpatient clinics and probably the most disabling feature of bilateral vestibulopathy in daily life. Moreover, disease onset was set as the timepoint symptoms were recognized by the patient for the first time, which might be unprecise, especially if the first examination took place many years after the subjective onset of complaints.

Together, our data encourage (i) screening for *RFC1* mutations not only in late‐ataxia cohorts but also in patients with isolated bilateral vestibulopathy as this approach possibly allows earlier detection of the disease, (ii) investigating distinct vestibular functions separately (semicircular canals, otoliths) as well as the vertical vestibular‐ocular reflex in a prospective study design as this might detect even earlier vestibular manifestations, and (iii) longitudinal analyses including comprehensive nerve conduction studies to decipher whether somatosensory neuropathy and bilateral vestibulopathy develop at the same time possibly reflecting that bilateral vestibulopathy is another manifestation of ganglionopathy in CANVAS (Palla et al., 2009).

## FUNDING

This study was funded by the Damp Foundation (to KL).

## COMPETING INTERESTS

The authors declare that they have no competing interests.

## AUTHOR CONTRIBUTIONS

MB, CH, and NB conceptualized the study. MB, VT, KL, CH, and NB contributed to data collection. KL performed genetic testing. Data analysis was performed by MB, IRK, and NB. MB drafted the first version of the manuscript. CH and NB contributed significantly to the writing of the manuscript. All authors critically revised and approved the final version of the manuscript.

### PEER REVIEW

The peer review history for this article is available at https://publons.com/publon/10.1002/brb3.2546

## Data Availability

The data used in this study are available from the corresponding author, upon reasonable request.
